# Elimination of HIV in South Africa through Expanded Access to Antiretroviral Therapy: A Model Comparison Study

**DOI:** 10.1371/journal.pmed.1001534

**Published:** 2013-10-22

**Authors:** Jan A. C. Hontelez, Mark N. Lurie, Till Bärnighausen, Roel Bakker, Rob Baltussen, Frank Tanser, Timothy B. Hallett, Marie-Louise Newell, Sake J. de Vlas

**Affiliations:** 1 Department of Public Health, Erasmus MC—University Medical Center Rotterdam, Rotterdam, Netherlands; 2 Nijmegen International Center for Health System Analysis and Education, Department of Primary and Community Care, Radboud University Nijmegen Medical Centre, Nijmegen, Netherlands; 3 Africa Centre for Health and Population Studies, University of KwaZulu-Natal, Mtubatuba, South Africa; 4 Department of Epidemiology, Warren Alpert Medical School, Brown University, Providence, Rhode Island, United States of America; 5 International Health Institute, Warren Alpert Medical School, Brown University, Providence, Rhode Island, United States of America; 6 Department of Global Health and Population, Harvard School of Public Health, Boston, Massachusetts, United States of America; 7 Imperial College London, London, United Kingdom; World Health Organization, Switzerland

## Abstract

**Background:**

Expanded access to antiretroviral therapy (ART) using universal test and treat (UTT) has been suggested as a strategy to eliminate HIV in South Africa within 7 y based on an influential mathematical modeling study. However, the underlying deterministic model was criticized widely, and other modeling studies did not always confirm the study's finding. The objective of our study is to better understand the implications of different model structures and assumptions, so as to arrive at the best possible predictions of the long-term impact of UTT and the possibility of elimination of HIV.

**Methods and Findings:**

We developed nine structurally different mathematical models of the South African HIV epidemic in a stepwise approach of increasing complexity and realism. The simplest model resembles the initial deterministic model, while the most comprehensive model is the stochastic microsimulation model STDSIM, which includes sexual networks and HIV stages with different degrees of infectiousness. We defined UTT as annual screening and immediate ART for all HIV-infected adults, starting at 13% in January 2012 and scaled up to 90% coverage by January 2019. All models predict elimination, yet those that capture more processes underlying the HIV transmission dynamics predict elimination at a later point in time, after 20 to 25 y. Importantly, the most comprehensive model predicts that the current strategy of ART at CD4 count ≤350 cells/µl will also lead to elimination, albeit 10 y later compared to UTT. Still, UTT remains cost-effective, as many additional life-years would be saved. The study's major limitations are that elimination was defined as incidence below 1/1,000 person-years rather than 0% prevalence, and drug resistance was not modeled.

**Conclusions:**

Our results confirm previous predictions that the HIV epidemic in South Africa can be eliminated through universal testing and immediate treatment at 90% coverage. However, more realistic models show that elimination is likely to occur at a much later point in time than the initial model suggested. Also, UTT is a cost-effective intervention, but less cost-effective than previously predicted because the current South African ART treatment policy alone could already drive HIV into elimination.

*Please see later in the article for the Editors' Summary*

## Abstract 2

Using nine structurally different models, Jan Hontelez and colleagues investigate timeframes for HIV elimination in South Africa using a universal test and treat strategy.

*Please see later in the article for the Editors' Summary*

## Editors' Summary

**Background:**

About 34 million people (mostly in low- and middle-income countries) are currently infected with HIV, the virus that causes AIDS, and every year another 2.5 million people become infected. HIV, which is usually transmitted through unprotected sex with an infected partner, gradually destroys CD4 lymphocytes and other immune system cells, leaving infected individuals susceptible to other infections. Early in the AIDS epidemic, people infected with HIV often died within ten years of infection. Then, in 1996, antiretroviral therapy (ART) became available, and, for people living in affluent countries, HIV/AIDS became a chronic condition. However, ART was expensive, so HIV/AIDS remained a fatal condition for people living in resource-limited countries. In 2006, the international community set a target of achieving universal ART coverage by 2010, and ART programs were initiated in many resource-limited countries. Although universal ART coverage has still not been achieved in South Africa, where nearly 6 million people are HIV-positive, 80% of people in need of ART were receiving a World Health Organization–recommended ART regimen by October 2012.

**Why Was This Study Done?:**

ART is usually started when a person's CD4 count falls below 350 cells/µl blood, but it is thought that treatment of all HIV-positive individuals, regardless of their CD4 count, could reduce HIV transmission by reducing the infectiousness of HIV-positive individuals (“treatment as prevention”). Might it be possible, therefore, to eliminate HIV by screening everyone annually for infection and treating all HIV-positive individuals immediately? In 2009, a mathematical modeling study suggested that seven years of universal test and treat (UTT) could eliminate HIV in South Africa. The deterministic (nonrandom) model used in that study has been widely criticized, however, and some subsequent modeling studies have reached different conclusions, probably because of differences in the models' structures and in the assumptions built into them. A better understanding of the reasons for the discrepancies between models would help policy-makers decide whether to introduce UTT, so, here, the researchers developed several increasingly complex and realistic models of the South African HIV epidemic and used these models to predict the long-term impact of UTT in South Africa.

**What Did the Researchers Do and Find?:**

The researchers developed nine structurally different mathematical models of the South African HIV epidemic based on the STDSIM framework, a stochastic microsimulation model that simulates the life course of individuals in a dynamic network of sexual contacts and in which events such as HIV infection are random processes. The simplest model, which resembled the original deterministic model, was extended by sequentially adding in factors such as different HIV transmission rates at different stages of HIV infection and up-to-date assumptions regarding the ability of ART to reduce HIV infectiousness. All the models replicated the prevalence of HIV in South Africa (the proportion of the population that was HIV-positive) between 1990 and 2010, and all predicted that UTT (defined as annual screening of individuals age 15+ years and immediate ART for all HIV-infected adults starting in 2012 and scaled up to 90% coverage by 2019) would result in HIV elimination (less than one new infection per 1,000 person-years). However, whereas the simplest model predicted that UTT would eliminate HIV after seven years, the more complex, realistic models predicted elimination at much later time points. Importantly, the most comprehensive model predicted that, although elimination would be reached after about 17 years of UTT, the current strategy of ART initiation for HIV-positive individuals at a CD4 cell count at or below 350 cells/µl would also lead to HIV elimination, albeit ten years later than UTT.

**What Do These Findings Mean?:**

These findings confirm previous predictions that UTT could eliminate HIV in South Africa, but the development of more realistic models than those used in the past suggests that HIV elimination would occur substantially later than originally predicted. Importantly, the most comprehensive model suggests that HIV could be eliminated in South Africa using the current strategy for ART treatment alone. As with all modeling studies, the accuracy of these findings depends on the assumptions built into the models and on the structure of the models. Thus, although these findings support the use of UTT as an intervention to eliminate HIV, more research with comprehensive models that incorporate factors such as data from ongoing trials of treatment as prevention is needed to determine the population-level impact and overall cost-effectiveness of UTT.

**Additional Information:**

Please access these websites via the online version of this summary at http://dx.doi.org/10.1371/journal.pmed.1001534.

- This study is further discussed in a *PLOS Medicine* Perspective by Ford and Hirnschall
- Information is available from the US National Institute of Allergy and Infectious Diseases on HIV infection and AIDS
- NAM/aidsmap provides basic information about HIV/AIDS and summaries of recent research findings on HIV care and treatment
- Information is available from Avert, an international AIDS charity, on many aspects of HIV/AIDS, including information on HIV and AIDS in South Africa, on HIV treatment as prevention and the possibility of HIV elimination (in English and Spanish)
- The 2012 UNAIDS World AIDS Day Report provides up-to-date information about the AIDS epidemic and efforts to halt it
- The World Health Organization provides information about universal access to AIDS treatment (in several languages); its 2010 ART guidelines can be downloaded
- The *PLOS Medicine* Collection Investigating the Impact of Treatment on New HIV Infections provides more information about HIV treatment as prevention
- Personal stories about living with HIV/AIDS are available through Avert, through NAM/aidsmap, and through the charity website Healthtalkonline

## Introduction

South Africa is home to the largest population of HIV-infected individuals worldwide, with nearly 6 million people living with HIV in 2010 [1]. Although extensive efforts to curb the epidemic may have resulted in some decline in the number of new HIV infections among young adults in the past few years [2],[3], incidence levels remain considerable. The proof of concept that antiretroviral therapy (ART) can be used to prevent onward transmission [4],[5], created renewed excitement that a turning point in the ever-growing HIV epidemic could be achieved by expanding access to treatment. “Treatment as prevention” (treatment of all HIV-infected individuals with ART, regardless of CD4 cell count, in order to reduce transmission)—a hypothesized HIV prevention intervention that is currently being tested in community randomized trials [6]—was conceptually designed by mathematical models [7]–[13]. In 2009, Granich et al. suggested that the HIV epidemic in South Africa could be driven into an elimination phase (defined as an incidence of below one new infection per 1,000 person-years) after just 7 y of annual HIV screening for individuals aged 15 y and older and immediate ART for all HIV-infected patients (universal test and treat [UTT]) [9].

In response to these results, other modeling studies also examined the potential impact of a UTT intervention in various settings [14]–[19]. But there are as many different conclusions as there are models that investigated the issue. As models are profoundly different in many aspects—structure, parameterization, and assumptions about the intervention—it is difficult to determine which factors are responsible for the differences in the model predictions [20]. There are several obvious reasons for these discrepancies, such as differences in the time horizon of the analysis [14], less or more optimistic assumptions regarding programmatic efficacy [14],[18], or different assumptions about HIV natural history, heterogeneity in transmission, and ART effectiveness in reducing infectiousness [17]. For example, Granich et al. assumed a 99.4% reduction in infectiousness of those on ART [9], but later studies suggested that this reduction is likely to be too optimistic [4],[21]–[23]. The HPTN 052 trial showed a reduction of 96% [4] with trial participants completely adhering to treatment, which is unlikely in large-scale interventions. A Cochrane review including all observational studies and the HPTN 052 trial reported a reduction in transmission of about 86% [21]. Also, the ongoing treatment rollout following the 2010 World Health Organization (WHO) treatment guidelines of ART at CD4 cell counts of ≤350 cells/µl [24] will already have a profound impact on the HIV epidemic [25],[26], making it important to compare the impact of UTT with the current treatment scale-up. Nevertheless, these obvious differences explain only part of the variation between model predictions [20]. As modeling remains essential to further inform public health decision-making, it is vital to better understand the reasons for the discrepancies between models.

**Figure 1:**
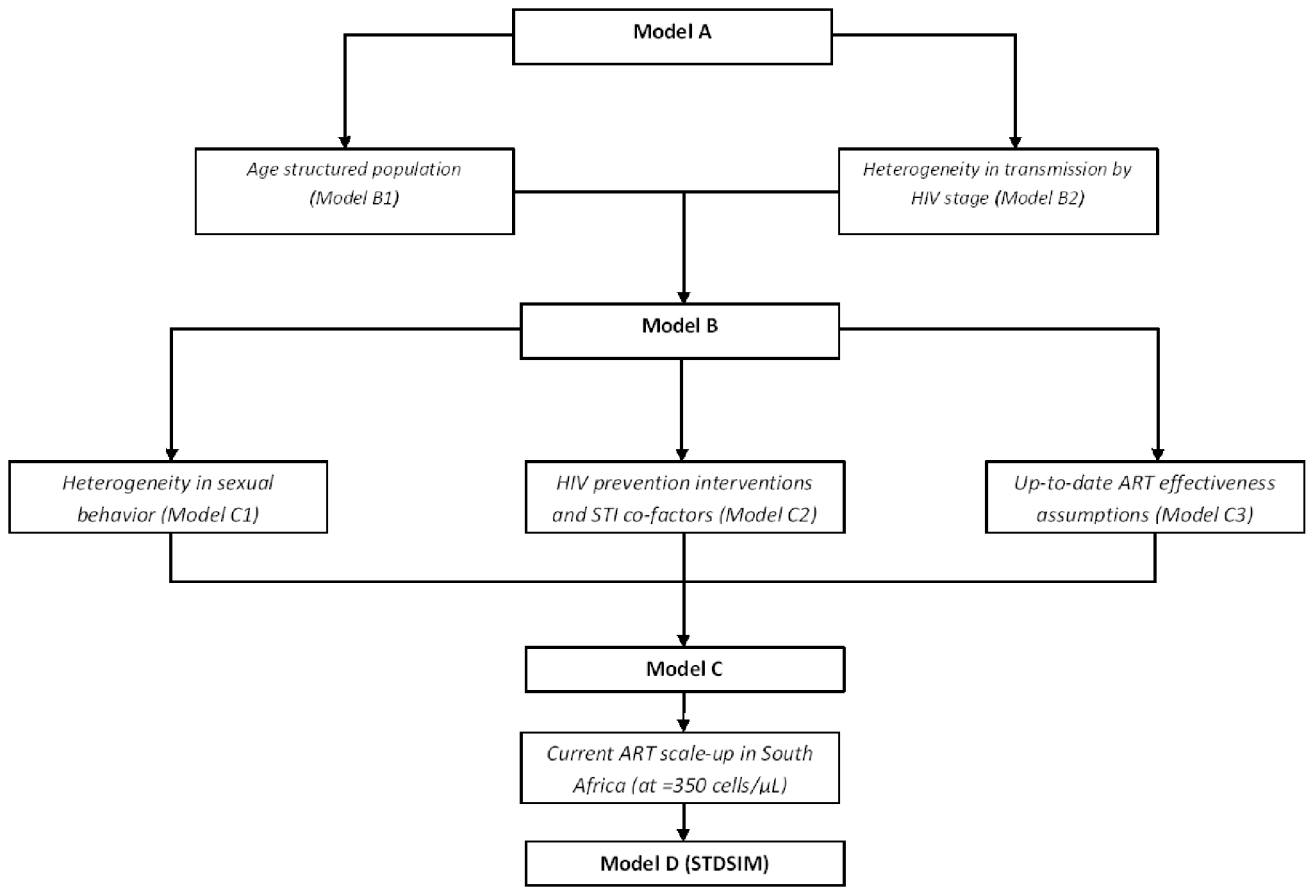
Stepwise approach of developing nine structurally different models with increasing complexity and realism. Model A resembles the deterministic model used by Granich et al. [9], now simulated using an event-driven approach. Models A and B are fitted to predict UNAIDS prevalence levels for South Africa by tuning the HIV transmission probabilities and year of HIV introduction. In addition, similar to Granich et al. [9], models A and B use a prevalence density function to explain the steady-state HIV prevalence observed in South Africa. Models C and D are fitted to represent UNAIDS-predicted HIV prevalence by adjusting overall partner change rates and the year of HIV introduction. A prevalence density function is no longer used, and the scaling-up of condom use in the late 1990s/early 2000s—introduced in model C2 and consistent with observations [2],[3]—is now used to explain the steady-state HIV prevalence in South Africa. Finally, models C and D allow for more realistic assumptions on the effectiveness of ART in reducing infectiousness (infectiousness reduction of 90% [20]–[22] instead of 99.4%; survival twice as high [26]).

We examined the impact of model structure and parameterization on the estimated impact of UTT in South Africa in a highly controlled experiment as follows: we developed nine structurally different models of the South African HIV epidemic with a standardized core set of assumptions but with gradated degrees of model complexity and realism that span from the very simplest to one of the most comprehensive representations of HIV epidemics (Figure 1). In all models, we examined the impact of the UTT intervention suggested by Granich et al. [9] and related this to a baseline of no UTT (i.e., no ART in the simplest models, and rollout of ART at CD4 count ≤350 cells/µl as currently applied in South Africa in the most detailed model).

## Methods

We developed four structurally different main models (models A, B, C, and D) and five sub-models (sub-models B1, B2, C1, C2, and C3) of the South African HIV epidemic through a stepwise approach of increasing complexity and realism (Figure 1; Table 1). With all models, we compare the impact of UTT to a no-UTT counterfactual (i.e., no ART in models A to C, and rollout of ART at CD4 count ≤350 cells/µl as currently applied in South Africa in model D). We used the STDSIM framework as a basis for all nine models. STDSIM is a stochastic microsimulation model that simulates the life course of individuals in a dynamic network of sexual contacts [27]. Events like partnership formation and the acquisition of infection are the result of random processes, determined by probability distributions. Therefore, the results are subject to stochastic variation, and the results in our study are based on the average of 1,000 runs.

**Table 1:** Overview of successive addition of components and structures to each of the nine models.

Component	Model A	Sub-Model B1	Sub-Model B2	Model B	Sub-Model C1	Sub-Model C2	Sub-Model C3	Model C	Model D
Prevalence density function^a^	X	X	X	X			X		
Age-structured population		X		X	X	X	X	X	X
Transmission probability by disease stage			X	X	X	X	X	X	X
Heterogeneity in sexual behavior					X			X	X
STI co-factors, male circumcision, and condom use						X		X	X
Up-to-date ART effectiveness assumptions							X	X	X
Current ART scale-up from 2003 onwards									X

a This artificial prevalence density function was introduced by Granich et al. [9] to mimic processes that result in the observed leveling off of the HIV epidemic. In the more comprehensive models of our analysis we replaced this prevalence density function by the actual processes that may be responsible for the leveling off.

The STDSIM model consists of four modules: demography, sexual behavior, transmission and natural history, and interventions. The demography module covers the processes of birth, death, and migration. Processes for initiation and dissolution of sexual relationships, mixing according to age preference, sexual contacts within relationships, and sexual contacts between female sex workers (FSWs) and their male clients are defined in the sexual behavior module. In the transmission and natural history module, transmission probabilities per sexual contact are specified for HIV and five other sexually transmitted infections (STIs): chancroid, chlamydia, gonorrhea, syphilis, and herpes simplex virus 2. The interventions module specifies the timing and effectiveness of control measures in curbing transmission (e.g., condom use) or enhancing survival (e.g., ART). STDSIM has been extensively used to evaluate behavioral interventions [28]–[31], syndromic treatment for STIs [32],[33], male circumcision [34], different HIV epidemics in sub-Saharan Africa [35], and, more recently, the impact of ART on HIV epidemics [25],[36]–[38]. STDSIM can be used in various levels of complexity by adding or removing components from the model. We exploit this flexibility to evaluate the test and treat strategy with alternative model structures.

### Model A

Model A fully resembles the deterministic model developed by Granich et al. [9], but now simulated using an event-driven stochastic approach. All individuals in the model constitute a homogeneous mixture of people in which HIV spreads from person to person. Individuals are assumed to have a one-off sexual contact with a random individual of the opposite sex every 8.5 d. We simulate a population aged 15–65 y with a constant background mortality rate of 0.025 per year. HIV is modeled in four consecutive stages with equal duration (30 mo) and transmission probabilities, and is introduced in the model by randomly “infecting” ten men and ten women. ART is assumed to decrease infectiousness by 99%, and the duration of the HIV stages for patients on ART is twice the duration of the stages for ART-naïve individuals [9]. In addition, as in the Granich model, transmission rates are further reduced by 40% because of simultaneous scale-up of other prevention interventions [9]. In accordance with Granich et al., we adjust transmission rates according to HIV prevalence through a prevalence density function to arrive at the observed steady state in HIV prevalence [9]: *p*(*t*_1_) = *p*(*t*_0_)×e^−*xP*^; where *p* = HIV transmission probability and *P* = HIV prevalence.

### Model B

To arrive at model B, we extended model A by adding the age-specific fertility and background mortality rates of South Africa (sub-model B1), and different stages of HIV disease progression (acute infection, asymptomatic infection, symptomatic infection, and AIDS—sub-model B2). Births are assigned randomly to sexually active women between the ages 15 and 49 y, and the probability of having a child depends on the age of the woman. We parameterized the model with Unite Nations–reported data on age-specific fertility rates for South Africa (Table S1) [39]. The resulting initial total fertility rate (defined as the expected lifetime number of births per woman) equals 4.9 births per woman. We reduced all age-specific fertility rates with equal factors in order to capture declines in fertility rates as observed in South Africa (Figure S1A) [39]. At birth, the age at (non-HIV) death of each individual is drawn from predefined sex-specific survival curves. In order to obtain background mortality rates in the absence of HIV for South Africa, we corrected the age- and sex-specific mortality rates for South Africa reported by WHO [40] using the cause-specific mortality estimates from WHO burden of disease estimates [41]. The resulting survival curve is shown in Figure S1B.

In sub-model B2, we added the following HIV disease progression: (i) early/acute infection with a median duration of 3 mo, (ii) asymptomatic infection with a median duration of 5 y, (iii) symptomatic infection with a median duration of 4 y, and (iv) AIDS with a median duration of 8 mo. All stages are exponentially distributed, and the resulting overall survival distribution has a median of 10 y and an interquartile range of 8 to 13 y, consistent with observations [42],[43]. Relative to asymptomatic infection (second stage), transmission probabilities are increased by a factor 15 during early/acute infection, a factor three during symptomatic infection, and a factor 7.5 during the AIDS stage [35],[44]. In addition, we assumed that the sexual activity of HIV-infected patients in the AIDS stage (last 40 wk of life) is reduced by 50% because of ill health [45].

### Model C

We extended model B with three components to arrive at model C. We added heterogeneity in sexual behavior (sub-model C1) by including different kinds of relationships. We use the same patterns of mixing as recently used by Hontelez et al. for a rural South African setting (the Hlabisa sub-district of KwaZulu-Natal) [25],[36],[37]. The model contains three types of sexual relationships: steady relationships, casual relationships, and commercial sex. The formation of partnerships occurs according to a supply-and-demand-based mechanism. People become available for a sexual relationship at an “age of sexual debut” that is randomly drawn at birth from a uniform distribution (Table S2). Each time the partnership status of a person changes (e.g., a partnership is formed or ended), a new duration until the person becomes available for a new relationship (“time until availability”) is drawn from a predefined exponential distribution with μ being the mean time until availability defined as: μ = τ*_s,r_*/(*r_s,a_*×*p*), with τ*_s,r_* = time interval by person's sex (*s*) and relationship status (*r*), *r_s,a_* = specific promiscuity factor by sex (*s*) and age (*a*), and *p* = personal promiscuity factor. The personal promiscuity factor (*p*) reflects the heterogeneity between individuals in the tendency to form partnerships, and is given by a gamma distribution with an average value (*p*_m_) of 1.0, and a shape parameter of 1.5 [35].

The duration of the availability period of an individual is given by an exponential distribution, with mean time to find (κ) defined as: 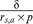, where the value of δ is 0.25 y for men and 2.25 y for women (Table S2) [30]. *r_s,a_* and *p* are explained above. When a person is available for a new relationship, he/she can be selected by an individual of the opposite sex who is at the end of his/her availability period. If a person has not been selected by the end of his/her availability period, he/she will select a partner from the pool of available persons of the opposite sex. The type of relationship (steady or casual) that is formed when a partner is selected depends on the age of the male partner, and is defined as the probability of a steady relationship (Table S2). The probability of a new relationship being a casual relationship is given by one minus the probability of a steady relationship. A relationship starts with a sexual contact. After each contact, the time until a new sexual contact *q* within the relationship is drawn from an exponential distribution with a mean frequency of sexual contact depending on relationship type and the age of the male partner (Table S2). Finally, the duration of a new relationship is drawn from an exponential distribution, where the average relationship duration depends on the relationship type (Table S2).

Partner selection at the end of the time to find κ is guided through an age preference matrix (Table S3), which defines the probability of selecting a partner from a certain age class. When there is no partner available in the preferred age class, immediate resampling is done of a new preferred age class using the remaining age groups with a probability larger than 0. If no partner can be found in any of the age classes, a new κ value is drawn from the above described equation. Probabilities in the age preference matrix are chosen to have men prefer slightly younger women. Preference matrices for both sexes are given in Table S3.

In the model, men can have sexual contacts with FSWs. A man's frequency of sexual contacts with a FSW is determined by defining frequency classes (in this study 0, 1, and 12 times per year [25],[35]). For each class, the proportion of men with and without a steady relationship falling in that category can be specified. A personal “prostitute-visiting inclination,” assigned to each male at birth, determines which individual men are assigned to which frequency classes. At sexual debut and at each sexual contact with a FSW, the next sexual contact with a FSW is scheduled according to an exponential distribution, with the mean duration until next contact based on the FSW contact frequency class of the individual.

The number of FSWs in the model results from male demand. New FSWs are recruited from sexually active women with a defined age range. The number of available FSWs and their predefined number of clients per week is checked each year and matched with the number of male contacts with FSWs. If the number FSWs is too low, new FSWs are recruited. If the number is too high, a random selection of FSWs terminate their career. For this study, we used the same values for number of FSWs and number of male contacts with FSWs as previously used for KwaZulu-Natal, South Africa [25] (Table S4).

In model C2, we added male circumcision (prevalence = 35% [46],[47]; reduces the risk of HIV acquisition by 50% [48]–[50]) and STIs that act as co-factors for HIV transmission (chlamydia, gonorrhea, chancroid, syphilis, and herpes simplex virus 2). Natural history and transmission assumptions for all STIs are described in Table S5. In addition, the model allows for increasing rates of condom use in the late 1990s/early 2000s, consistent with observations [2],[3], to replace the prevalence density function used in models A and B.

Sub-model C3 uses more up-to-date assumptions regarding the effectiveness of ART in reducing HIV transmission and enhancing survival. Granich et al. assumed that ART reduces infectiousness by 99% [9]. However, recent observational studies and a randomized controlled trial showed that effectiveness is likely to be lower [21]–[23]. A meta-analysis of observational studies found an average reduction of 92% [22], which was later also reported by Donnell et al. [23]. The HPTN 052 trial showed a reduction of 96% [4]; however, the participants in the trial were completely adherent to treatment, which is unlikely in large-scale interventions. A Cochrane review including all observational studies and the HPTN 052 trial reported a reduction in transmission as a result of ART of about 86% [21]. Here, we assume a 90% efficacy in reducing transmission, implicitly allowing for imperfect adherence. In addition, Granich et al. assumed that ART increases survival of HIV-infected patients by a factor of two relative to their remaining life expectancy without HIV treatment. We assume the survival benefit of ART to be twice as high as assumed by Granich et al. (i.e., a factor four increase), consistent with observations [51].

### Model D

Model D resembles the full STDSIM model [25],[35]–[38], consisting of all of the features in model C but with two important additions: CD4 cell count decline during disease progression and the ART rollout in South Africa for the period 2004–2011. Based on data from Orange Farm, South Africa, we assumed that the initial (HIV-negative) CD4 cell count in the population follows a lognormal distribution with median 7.02 (equivalent to 1,116 cells/µl) [52]. CD4 cell counts decline by 25% during acute infection, and decline linearly over the other stages until the CD4 cell count reaches 0.5% of its initial value, after which an individual dies of AIDS [9],[25],[52]. Therefore, based on average HIV-negative CD4 cell counts and duration of HIV infection, an individual will, on average, reach a CD4 cell count of ≤350 cells/µl about 6 y following infection, and at that point will have a remaining life expectancy of about 4 y.

In the model, ART coverage is the result of two components: (i) an individual's demand for ART as a function of HIV disease stage, and (ii) the capacity of the health system to meet this demand. ART coverage in our model is the ART demand met by the capacity of the health system. We assumed the ART demand function to be the same as previously estimated for the Hlabisa sub-district of KwaZulu-Natal, South Africa [25], in which about 30% of all infected individuals first seek care for HIV well after their CD4 cell count has dropped below 200 cells/µl. We fitted the model predictions to observed ART coverage levels over the period July 2004–July 2010 by performing a grid search on three parameters: start year of ART scale-up, rate of ART scale-up, and ART scale-up function (three options: linear, square-root, or quadratic). We optimized predicted ART coverage by calculating the mean squared error of predicted ART coverage in the model compared to coverage data reported by WHO [53]. We assumed that eligibility criteria changed from ART at CD4 count ≤200 cells/µl to ART at CD4 count ≤350 cells/µl in August 2011, and that ART scale-up continued according to the estimated scale-up pattern for the years 2012–2050 in the baseline.

### Model Fitting and Parameter Uncertainty

We fitted all models to replicate the observed HIV prevalence in South Africa as reported by the Joint United Nations Programme on HIV/AIDS (UNAIDS) [1]. For each model, we used the parameters of three aspects that link directly to three different characteristics of the HIV epidemic in South Africa: (i) the start of the epidemic, (ii) the growth of the epidemic over the 1990s, and (iii) leveling off of the epidemic in the early 2000s. We obtained the best fit by minimizing the squared errors of predicted and reported HIV prevalence over the period 1990–2010.

We used the following parameters to fit the models/sub-models A, B1, B2, B, and C3: (i) year of HIV introduction, (ii) transmission probabilities in men with asymptomatic HIV; (iii) the *x* parameter in the prevalence density function. We fitted sub-model C1 in 2 parts: (i) the initial growth of the epidemic in the 1990s, and (ii) the leveling off of the epidemic in the 2000s. For step 1, we fitted three parameters: year of HIV introduction, HIV transmission probabilities in individuals with asymptomatic HIV, and relative partner change rates (multiplying all values of sex- and age-specific promiscuity—see above—by a certain factor). For step 2, we assumed changes in the observed leveling off of the HIV epidemic to be the result of behavior change—defined as changes in relative partner change rates—and used three parameters: year of start of behavior change, rate of change in behavior change, and end year of behavior. The sexual behavior pattern resulting from the best fit by sex and age for the year 1990 (start of the epidemic) is given in Figure S2, and for the year 2003 (after above described reduction in risk behavior) in Figure S3. We used the following parameters to fit sub-model C2 to observed HIV prevalence levels: (i) year of introduction of HIV, (ii) transmission probabilities in individuals with asymptomatic HIV, and (iii) scale-up of condom use over time. We assume a stepwise linear increase of condom use over time, and estimate the start year, slope, and end year of the scale-up that results in the best-fitting HIV prevalence. For models C and D, we fixed the HIV transmission probability in individuals with asymptomatic HIV at 0.001 for men and 0.0005 for women [12]. In order to start the epidemic, we introduced HIV in six FSWs. We used the following parameters to fit the model: (i) year of HIV introduction, (ii) relative partner change rates, and (iii) start year, rate, and end year of condom use scale-up in casual relations and commercial sex. The best fit for model D resulted in a 30% condom use rate in casual relationships, which is comparable to data [54].

We calculated a range around the baseline estimates reflecting the uncertainty in the parameters that were used to fit all nine models. We developed uniform distributions with intervals wide enough to capture all possible parameter values that could produce a good fit (defined as a predicted HIV prevalence within the uncertainty interval provided by UNAIDS for the period 1995 to 2010 [1]), and randomly drew values from these distributions to serve as model input. We ran the model with these parameter values, and accepted parameter value combinations only when they produced a good fit. For all parameter value combinations for which this was the case, we recalculated all the outcomes of the study.

For model D (the most comprehensive model in our analysis) we repeated this procedure until we arrived at 120 alternative parameter values that were accepted based on the above mentioned criterion, and calculated all main outcomes of this study with these alternative parameter settings. We developed ranges reflecting parameter uncertainty by discarding the three highest and lowest values for each outcome. For all other models and sub-models, we repeated the procedure until we arrived at 40 alternative parameter values that were accepted, and discarded only the single highest and lowest outcome values in order to crudely arrive at the interval. We incorporated the intervals resulting from this parameter uncertainty analysis as bars and ranges in graphs and tables. A visual representation of all parameter combinations that were used to calculate uncertainty ranges, including the combinations that produced the highest and lowest values that were later discarded, is given in Figure S4.

### Scenarios and Outcomes

For all nine models we predicted the impact of a hypothetical UTT intervention with annual screening of individuals aged 15+ y and immediate ART for those who are infected with HIV, as modeled by Granich et al., i.e., the intervention is scaled up linearly to 90% coverage in seven years' time (2012–2019), and there is a dropout rate of 8.5% in the first year of treatment and 1.5% in subsequent years [9]. We assume no further scale-up of other prevention interventions (e.g., condom use, circumcision) after 2012 in all models. Following Granich et al. we defined the “elimination phase” of HIV to start when HIV incidence drops below 1 new infection per 1,000 person-years [9].

Furthermore, we calculated the cumulative number of life-years saved and cumulative net costs of UTT compared to continued scale-up of ART at CD4 count ≤350 cells/µl in model D, the only model that incorporates enough detail to be able to adequately represent the current scale-up of ART at CD4 count ≤350 cells/µl in South Africa as the baseline of no UTT. We analyzed costs from the health care sector perspective, and assumed ART costs similar to those in Hontelez et al. [25]—where annual ART costs were stratified by CD4 cell count at initiation and thereafter dependent on the number of years on treatment (Table 2)—which were derived from ART programs in Cape Town, South Africa [55],[56]. Costs include costs for ART provision, treatment of opportunistic infections, outpatient visits, and inpatient days. Costs were stratified by CD4 cell count at initiation since those initiating treatment at late stages (low CD4 cell count) are more likely to have opportunistic infections and other complications, thus requiring more additional care. This difference disappears after subsequent years of successful treatment. We assumed annual ART costs for those initiating ART at CD4 cell counts of >350 cells/µl to be similar to the costs for those who initiated at 200–350 cells/µl and were on treatment for more than 2 y. For the UTT scenario, in which treatment initiation is not guided by CD4 cell count, we lowered all cost input values in the first year of treatment by US$104 to subtract the cost of a CD4 cell count test [25]. We discounted future costs and life-years saved by 3% annually [57].

**Table 2:** Cost input values used in this study.

CD4+ Count (in Cells/Microliter) at ART Initiation	Per Patient Annual ART Costs (US Dollars)		
	First Year	Second and Third Year	Subsequent Years
0–100	3,664	1,435	1,095
101–200	3,060	1,284	1,095
201–350	2,304	1,095	1,095
>350	1,095	1,095	1,095

Costs are stratified by CD4+ count at ART initiation, and include costs of diagnostic testing, ART provision, treatment of opportunistic infections, outpatient visits, and inpatient days.

Finally, we performed a sensitivity analysis on all the results from model D by varying parameters of HIV natural history, heterogeneity in HIV transmission, the state of the HIV epidemic in South Africa, more and less optimistic UTT intervention outcomes, and the assumptions on overall cost and scale effects. Details on the sensitivity analysis can be found in Text S1.

## Results

**Figure 2:**
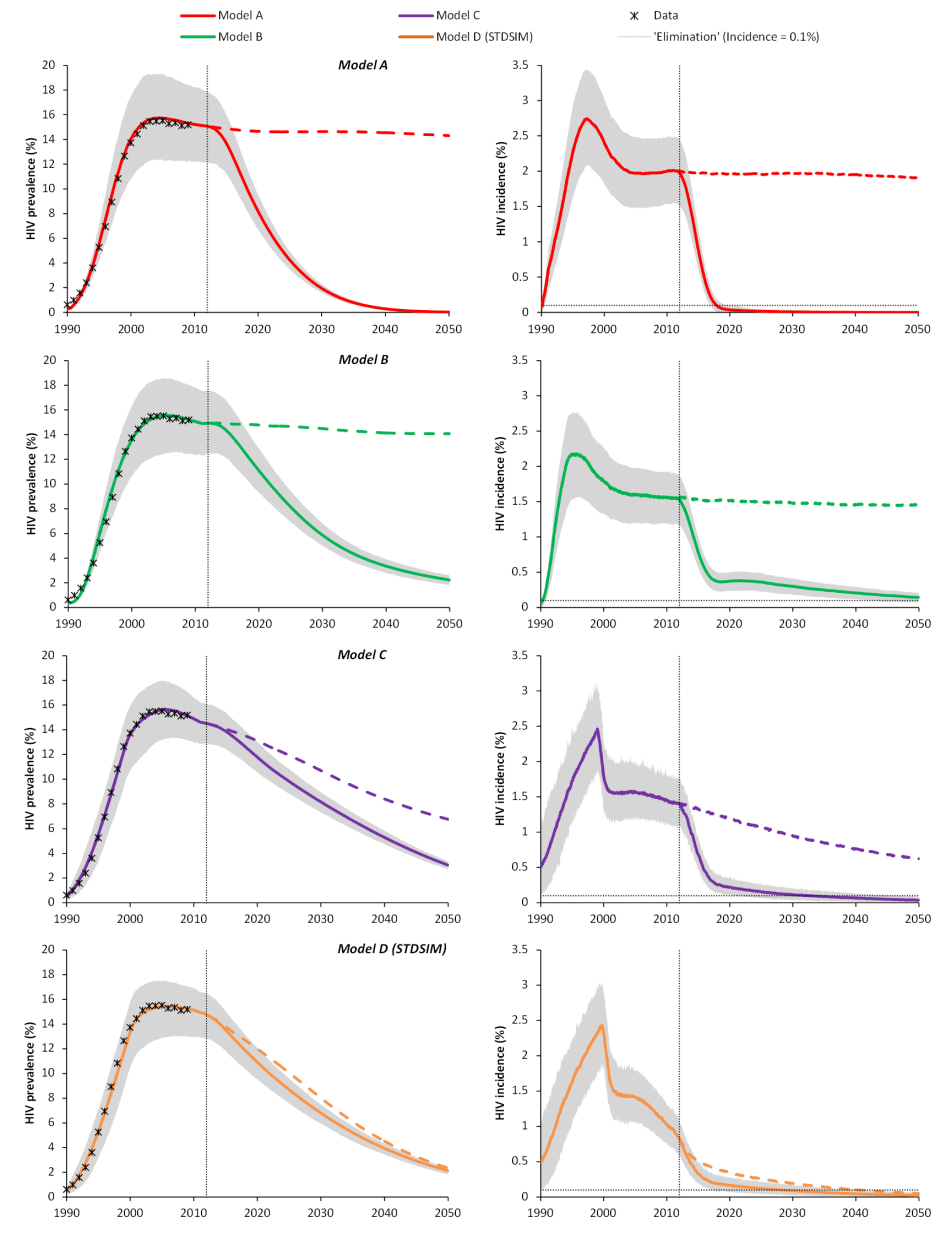
Predicted impact of universal testing and immediate ART for all HIV-infected patients (UTT) on HIV prevalence and incidence in adults (aged 15+ y) for four main models of the South African HIV epidemic over the period 1990–2050. Left panels: HIV prevalence; right panels: HIV incidence. All models are structurally different. Solid lines represent the impact of the UTT intervention; the dashed lines represent the no-UTT counterfactual. Colored lines are the average result of 1,000 simulations, and the gray areas represent the probability intervals illustrating 95% of the stochastic variation around the baseline estimate. UTT is implemented as annual screening of the adult population (aged 15+ y), and immediate ART for all HIV-infected patients. The intervention is scaled up linearly, starting in 2012 and reaching 90% coverage in 2019 (similar to Granich et al. [9]). The vertical dotted lines give the timing of the start of the intervention. The horizontal dotted lines in the right panels indicate the elimination phase, defined as incidence below 1/1,000 person-years. Structures and components of the different models are explained in Figure 1.

Figure 2 (left panels) shows the fit of all models to the HIV prevalence in South Africa as reported by UNAIDS [1], together with the projected impact of annual screening of individuals aged 15+ y and immediate ART for all HIV-infected patients at 90% coverage. All models replicate the HIV prevalence in South Africa in the period 1990–2010. However, as a result of the difference in underlying processes in the structurally different models, the corresponding HIV incidence levels are substantially different (Figure 2, right panels). For example, the predicted incidence in 2011 for model A was 2.0/100 person-years, while for model D this was only 1.0/100 person-years (right panels of Figure 2). Also, projections regarding the future course of the HIV epidemic in the absence of UTT differ substantially. Future incidence and prevalence in the absence of treatment reach a steady state in models A and B, as indicated by the dashed lines. In model C, the incidence and prevalence of HIV already decline in the no-intervention scenario because of the increase in condom use in the early 2000s. Such a decline is even more profound in model D, where current ART scale-up in South Africa is included.

All models are consistent in predicting that HIV will eventually be eliminated by UTT. However, the timing of elimination significantly differs between the models (Figure 2; Table 3). In model A, the HIV epidemic is driven into an elimination phase after 7 y (range: 6; 9), while in models B, C, and D the elimination phase is reached only after 39 (range: 30; 49), 21 (range: 19; 30), and 17 (range: 15; 23) y, respectively. For model D, the HIV incidence is even projected to reach the elimination phase in 2041 (range: 2037; 2047) without the full UTT intervention, because of the impact of the current scale-up of ART at CD4 cell counts of ≤350 cells/µl.

**Table 3:** Year of HIV elimination (incidence <1/1,000 person-years) under universal testing and immediate ART for all HIV-infected patients (UTT) and number of life-years saved through UTT compared to the baseline of no UTT.

Model	Sub-Model	Year of Elimination^a^ (Range)	Life-Years Saved per ART Treatment Year in 2050 (Range)
**Model A**		2019 (2018; 2021)	5.7 (4.7; 7.2)
	+ Age structure (B1)	2019 (2018; 2020)	3.8 (3.1; 4.3)
	+ Heterogeneity in HIV transmission by disease stage^b^ (B2)	2053 (2048; >2060)	2.6 (2.1; 3.3)
**Model B (B1 and B2 combined)**		2053 (2042; >2060)	3.0 (2.6; 3.5)
	+ Sexual network (C1)	>2060 (2058; >2060)	2.6 (1.8; 2.9)
	+ Background prevention interventions (C2)	2042 (2037; 2050)	2.8 (2.1; 3.2)
	+ Up-to-date ART assumptions (C3)	>2060 (2054; >2060)	2.9 (2.5; 3.1)
**Model C (C1, C2, and C3 combined)**		2032 (2030; 2041)	1.8 (1.1; 2.0)
**Model D (STDSIM)**		2029 (2027; 2034)	1.7 (1.2; 2.6)
**Model D baseline (ART at CD4 count ≤350 cells/µl)**		2041 (2037; 2047)	N/A

UTT is scaled up linearly, starting in 2012 and reaching 90% coverage in 2019. Ranges reflect the variation in outcome due to the uncertainty in the parameter values that were quantified based on fitting the model to the data.

a Incidence below 1/1,000 person-years.

b We assumed four different stages: acute, asymptomatic, symptomatic, and AIDS.

N/A, not applicable.

The sub-models B2, C1, and C3 do not predict elimination of HIV by 2050 (Table 3; Figure S5). The combination of a high background mortality and heterogeneity in HIV transmission (model B2) results in a disproportionate contribution of acute infection to the overall epidemic (as many HIV-infected patients die because of other causes), thereby limiting the potential impact of UTT. Heterogeneity in sexual behavior also prolongs the predicted time to elimination (sub-model C1), as it accounts for high-risk individuals who continue to spread HIV even in the presence of ART. Finally, it is obvious that more up-to-date assumptions on ART effectiveness result in a lower predicted impact of UTT (sub-model C3), as the reduction in infectiousness is lower (90% versus 99.4%) and survival is higher (twice as high compared to Granich et al. [9]). On the other hand, explicitly modeling background prevention interventions and STI co-factors (sub-model C2) instead of using a simple prevalence density function shortens the time until elimination, as the interventions scaled up before 2012 affect the dynamics of the epidemic in the long run, reducing incidence even without UTT or further scale-up of other interventions (dashed line for model C in Figure 2).

**Figure 3:**
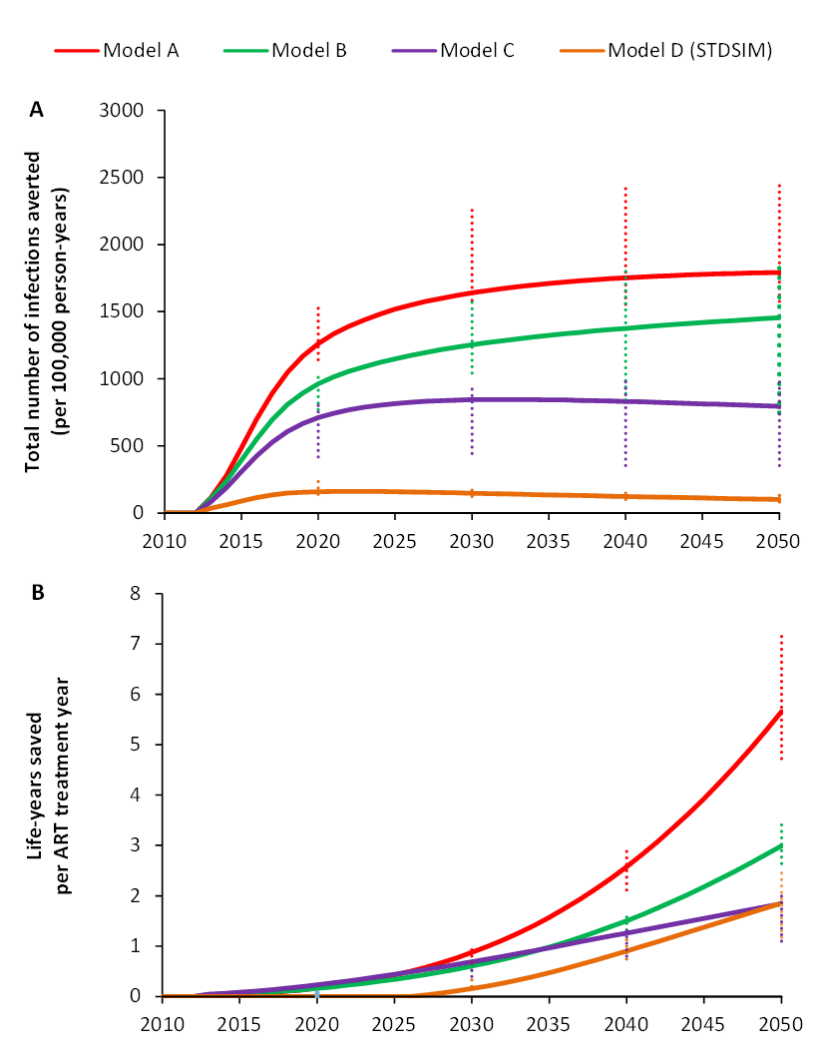
Number of infections averted per 100,000 person-years and cumulative number of life-years saved per ART treatment year for universal testing and immediate ART for all HIV-infected patients (UTT) in South Africa over the period 2010–2050. The intervention consists of annual screening of the adult population (aged 15+ y), and immediate ART for all HIV-infected patients. Intervention is scaled up linearly starting in 2012 and reaching 90% coverage in 2019. (A) Difference between cumulative numbers of new infections per 100,000 person-years in the UTT intervention scenario versus the baseline (for models A, B, and C, the baseline is no ART; for model D, the baseline is ART at CD4 count ≤350 cells/µl). (B) Cumulative number of life-years saved per person-year on ART treatment in the UTT intervention compared to the baseline (for models A, B, and C, the baseline is no ART; for model D, the baseline is ART at CD4 count ≤350 cells/µl). Error bars reflect ranges due to the uncertainty in the parameter values that were quantified based on fitting the model to the data.

There are substantial differences in the impact of UTT compared to the no-UTT baseline in the different models, which has important consequences for the effectiveness of the intervention. By 2050, a cumulative total of 1,800 new infections per 100,000 person-years (range: 1,600; 2,500) would be averted in model A, while this value is only 100 (range: 83; 150) in model D (Figure 3A). Consequently, the predicted efficiency of ART in saving lives also differs substantially between models (Figure 3B). Model A predicts about 5.7 cumulative life-years saved per treatment year by 2050 (range: 4.7; 7.2), while in model D this value is only 1.7 (range: 1.2; 2.6), almost four times lower.

**Figure 4:**
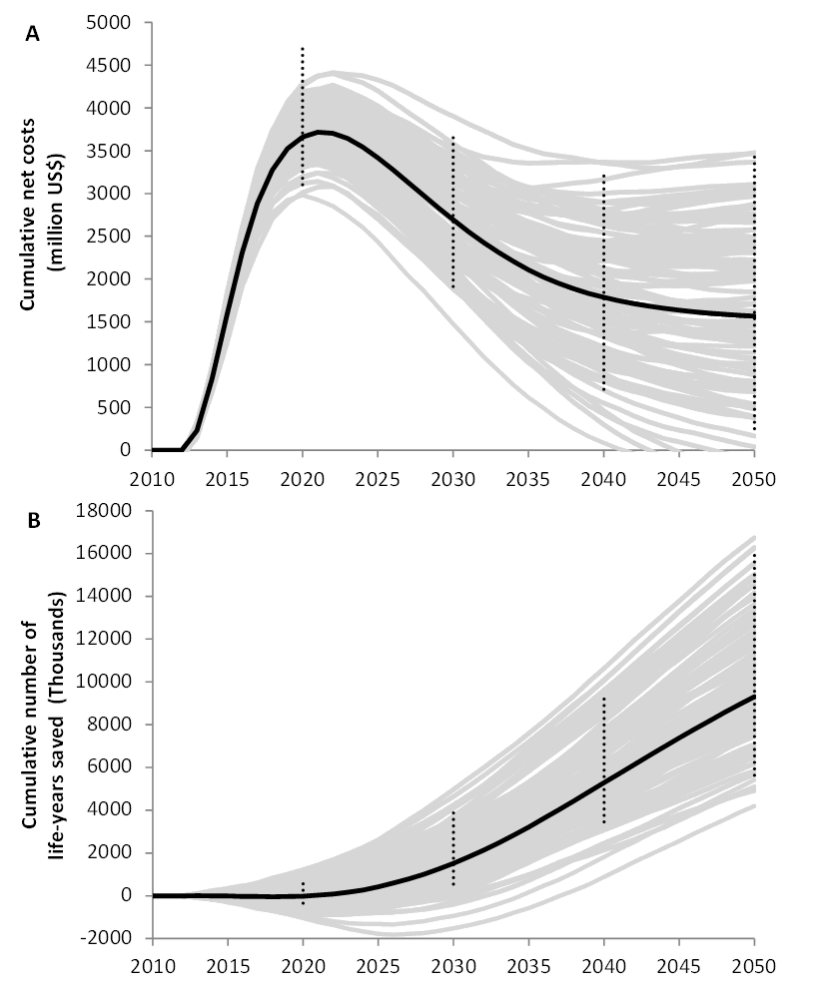
Cumulative net costs and cumulative number of life-years saved of universal testing and immediate ART for all HIV-infected patients (UTT) compared to the current rollout in South Africa of ART at CD4 count ≤350 cells/µl, as predicted with model D. (A) Cumulative net costs; (B) cumulative number of life-years saved. Grey lines represent 100 individual model runs illustrating stochastic variation; black lines are averages over 1,000 model runs. Error bars reflect ranges due to uncertainty in the parameter values that were quantified based on fitting the model to data. Costs and life-years saved were discounted at an annual rate of 3%.

Model D shows that cumulative net costs peak at US$3.8 billion (range: 3.1 billion; 4.7 billion) at around 2020, and decline thereafter as the impact of UTT on HIV incidence is translated into a lower number of patients on treatment (Figure 4A). Cumulative net costs reach about US$1.8 billion in 2050 (range: 0.25 billion; 3.5 billion). The effects of UTT will become apparent only around the year 2020, when the prevented infections translate into life-years saved (Figure 4B), and life-years saved increase linearly to 10.4 million by 2050 (range: 5.6 million; 16.1 million). The resulting incremental cost-effectiveness ratio is US$170/life-year saved (range: 19; 406) (Table 4). All results are robust to alternative assumptions and parameterizations (see sensitivity analysis in Text S1 and Tables S6, S7, S8).

**Table 4:** Effects, cost, and cost-effectiveness of universal test and treat versus continued scale-up of ART at CD4 count ≤350 cells/µl in South Africa over the period 2012–2050 (Model D).

Strategy	Cumulative Life-Years (Millions) (Range)	Δ Life-Years (Millions) (Range)	Cumulative Costs (Millions of US Dollars) (Range)	Δ Cost (Millions of US Dollars) (Range)	Incremental Cost-Effectiveness Ratio (US Dollars/Life-Year Saved) (Range)
ART at CD4 count ≤350 cells/µl	1,290 (1,200; 1,360)	—	76,900 (67,700; 90,100)	—	—
UTT	1,300 (1,210; 1,380)	10.4 (5.6; 16.1)	78,600 (69,100; 93,200)	1,780 (250; 3,470)	170 (19; 406)

Costs and effects are discounted at an annual rate of 3%. Life-years concern the total life-years lived in South Africa of the entire population. Of these total life-years, 7% are life-years lived with HIV. Ranges reflect the variation in outcome due to the uncertainty in the parameter values that were quantified based on fitting the model to the data.

## Discussion

Our study confirms previous reports that an intervention of universal voluntary counseling and testing for individuals aged 15+ y and immediate ART for all HIV-infected individuals (UTT) at 90% coverage will eventually result in the elimination of HIV, even in a highly endemic setting such as South Africa and with realistic assumptions about the efficacy of ART in reducing HIV transmission and enhancing survival. However, the predicted timing of the elimination of HIV (defined as an incidence of below one new infection per 1,000 person-years) differs substantially for the different models in our study, and HIV elimination is likely to take three times longer than the mere 7 y predicted by Granich et al. [9]. In addition, the relative impact of the UTT intervention compared to the baseline differs substantially. Whereas 1,800 infections are averted per 100,000 person-years (range: 1,600; 2,500) in the simplest model, this value is only 100 (range: 83; 150) in the most comprehensive model. In fact, the latter model shows that the current scale-up of ART for patients with CD4 cell counts of ≤350 cells/µl already leads to elimination of HIV without the additional UTT intervention. However, the considerable number of life-years saved makes UTT at 90% coverage still a highly cost-effective intervention, with an incremental cost-effectiveness ratio of US$170/life-year saved (range: 19; 406).

Our sub-model analysis shows that choices in model structure and assumptions have an important impact on the predicted impact of UTT. It makes sense that more up-to-date assumptions on the overall efficacy of ART in reducing infectiousness (90% versus 99.4%) lead to delayed HIV elimination. Also, incorporating high infectiousness during the acute stage results in a less profound impact of UTT, since relatively many transmission events will then occur during this short period of high infectiousness, which is difficult to target in UTT interventions [17],[58]. Adding heterogeneity in sexual behavior and sexual networks to the model also increases the time until elimination—this is because the relative force of infection of HIV is high in certain high-risk groups (e.g., FSWs) and therefore the impact of UTT is less profound in these subgroups. Finally, explicitly modeling male circumcision, condom use, and STI co-factors, and using increases in condom use to quantify the HIV epidemic in South Africa [2],[3], decreases the time until HIV elimination, since the counterfactual of no UTT already shows a substantial decline in incidence, despite the fact that these interventions are not further scaled up in the model after 2012. A model that relies on implicit modeling of these interventions to capture the steady state (e.g., through a prevalence density function, as was used by Granich et al. [9]) will therefore overestimate the impact of UTT. Finally, it appears vital to incorporate the current ART rollout in the counterfactual scenario. The availability of ART in South Africa and many other African countries is now a fact of life, and the rollout that generally started in 2003–2004 is already affecting the epidemics through increased survival and decreased transmission [2].

Given that the stepwise inclusion of model components appeared to change the predicted impact of a UTT intervention, the model that incorporates all these components (model D) gives the most accurate prediction of the impact. In addition, although all models were able to accurately replicate the UNAIDS-reported HIV prevalence in South Africa, model D was the only model that was also able to capture the observed decline in incidence over the past decade [2],[3]. In model D, incidence in the population aged 15–49 y declined from 1.9/100 person-years in 2002 to 1.3/100 person-years in 2008, which is nearly the same as the observed reduction from 2.0/100 person-years in 2002–2005 to 1.3/100 person-years in 2005–2008, as reported by Rehle et al. [3]. Incidence rates in the other models remained constant over the same period (models A to C). In addition, model D was able to replicate data on demographic structure, age-specific HIV prevalence, sexual behavior, STI prevalence, and ART coverage in South Africa (Figure S7; Text S2). Finally, previous studies with STDSIM have shown that the model is capable of reproducing HIV prevalence (overall and age- and sex-specific), incidence, and mortality data from a population-based HIV and demographic surveillance site in KwaZulu-Natal, South Africa [25],[36],[37],[59],[60]. The simulated impact of ART in this highly endemic area of South Africa [25] was very similar to what was recently observed by Tanser et al. [5], providing reassurance that our model predictions are accurate.

To our knowledge, this is the first study that shows that the current rollout of ART for all HIV-infected patients with CD4 cell counts of ≤350 cells/µl will eventually eliminate HIV. This raises questions about the value for money of the additional investments required to implement UTT. Although we show that the UTT intervention proposed by Granich et al. [9] is highly cost-effective, the required number of health workers and financial resources for such a strategy far exceeds the current availability in South Africa [61]. Also, the assumed rates of HIV testing, ART uptake, retention in care, and treatment adherence are rather optimistic [62],[63]. Adherence and retention are likely to decrease when treatment is initiated at higher CD4 cell counts [64], while the number of patients lost to follow-up increases when treatment programs are scaled up [65]. Both these issues are especially important in UTT strategies, where patient numbers increase substantially, and many patients initiate ART at high CD4 cell counts. In addition, maintaining screening coverage levels at 90% for 40+ y seems not very plausible. It is likely that test refusal will be substantially higher than the 10% assumed in our analyses [66], increase over time [67], and be more common among people with HIV [67], resulting in a lower and declining screening coverage over time. Still, our sensitivity analysis shows that UTT would remain a cost-effective strategy, even with coverage rates of only 60%. A recent study on the cost-effectiveness of ART provision in South Africa showed that cost savings will be achieved after just 5 y of UTT at 90% coverage [68], while our modeling indicates that there will be no net savings from this UTT intervention in South Africa. The underlying compartmental transmission model in that study [68] is essentially the same as that previously used by Granich et al. [9],[68], and thus resembles our model A. We show that these types of models, which ignore sexual networks and background prevention interventions underlying the current South African epidemic, predict a far more optimistic impact of UTT compared to the baseline. Cost-effectiveness and economic impact studies based on such models should therefore be interpreted with caution. More research with comprehensive models of the impact of more modest UTT interventions is necessary in order to determine whether universal treatment for HIV really is a cost-effective intervention.

Our study has a number of limitations. We defined elimination as incidence below 1/1,000 person-years. However, real elimination is achieved when both incidence and prevalence reach 0%. Microsimulation allows for such an analysis, and we found that in a model population of about 35,000 people, by 2080, 99% of all model runs predict that HIV prevalence reaches 0% in model A (Figure S5). In model D this point is reached only in year 2116 for the UTT scenario, and in year 2164 for continued scale-up of ART at CD4 count ≤350 cells/µl (Figure S5). In addition, we did not model the development and transmission of drug-resistant strains. Both acquired resistance (development of resistance within an individual on treatment) and transmitted resistance (spread of drug-resistant strains) will have an impact on the effectiveness of treatment programs, and will consequently result in a less profound effect of the current ART scale-up or UTT in South Africa. It is currently unclear, however, to what extent the fears of rapidly spreading drug resistance expressed at the start of the ART scale-up were justified [69]. The prevalence of drug resistance remains low in South Africa after nearly 10 y of scaling up ART [70],[71]. In addition, adherence to treatment is as high as in many high-income countries [72], and survival of patients on treatment in sub-Saharan Africa approaches general life expectancy [73], suggesting that resistance may not become a major problem in South Africa in the near future.

Elimination of HIV in South Africa will have huge implications for public health and socioeconomic development in the country. The current ART rollout is already resulting in a substantial increase in the life expectancy of the general population [74] and in the employment rates of HIV-infected people [75]. Finally, elimination of HIV will also substantially reduce the tuberculosis burden in South Africa, given the close link between the two epidemics [76].

In conclusion, our results from a series of structurally different models support the main message from previous studies that HIV in South Africa can be eliminated through a strategy of annual screening of individuals aged 15+ y and immediate ART for all HIV-infected patients at 90% coverage, but elimination will occur substantially later than previously predicted. Importantly, the most comprehensive model suggests that HIV incidence in South Africa can reach the elimination phase even if the current treatment scale-up of ART at CD4 count ≤350 cells/µl continues without the addition of a UTT intervention. Results from upcoming community randomized trials of treatment as prevention will need to be evaluated with models that allow for sufficient detail in assumptions in order to adequately project the population-level impact and overall cost-effectiveness of the UTT intervention.

## Supporting Information

Figure S1 **Demographic input parameters.** (A) Total fertility rates (lifetime number of births/woman) over time. Data obtained from the UN World Fertility Data 2008 database [39]. (B) Background mortality (mortality in the absence of AIDS) obtained from WHO [40]. All cause mortality rates were corrected using WHO burden of disease estimates of HIV mortality rates in South Africa [41] in order to obtain estimates of non-AIDS-related mortality rates in South Africa.
(TIF)

Figure S2 **Distribution of number of partners (steady and casual) in the last 12 mo by age and sex.** Results follow from parameter settings described in Tables S2–S4.
(TIF)

Figure S3 **Distribution of number of partners in the last 12 mo (steady and casual) by age and sex in 2003 (after change in overall partner change rates).** Results follow from parameter settings described in Tables S2–S4 and above described adjustment in overall partner change rates.
(TIF)

Figure S4 **Parameter combinations used to create uncertainty ranges around the baseline estimates for models A to D.** Because of underlying structural differences, different parameters were used to fit the models to UNAIDS-reported HIV prevalence data. Our approach to developing parameter combinations and uncertainty ranges is described in the Methods (“Model Fitting and Parameter Uncertainty”). Light-blue dots and lines indicate parameter combinations that produced the highest and lowest estimates of impact; these estimates were omitted to create 95% uncertainty ranges. For models A and B, we used the HIV introduction year and HIV transmission probabilities (left panels) and the *x* parameter in the prevalence density function *p*(*t*_1_) = *p*(*t*_0_)×e^−*xP*^, where *p* = HIV transmission probability and *P* = HIV prevalence (right panels), to fit the model. For models C and D, we used the relative partner change rates and introduction year of HIV (left panels) and the increase in condom use rates (right panels).
(TIF)

Figure S5 **Cumulative distribution of year of achieving 0% incidence and prevalence of individual model runs for all main models.** Results are based on 400 model runs.
(TIF)

Figure S6 **Predicted impact of universal testing and immediate ART for all HIV-infected patients (UTT) on HIV prevalence (left panels) and incidence (right panels) in adults (aged 15+ y) for five sub-models of the South African HIV epidemic over the period 1990–2050.** Colored lines are the average result of 1,000 simulations, and the grey areas represent the probability intervals based on the stochastic variation between individual model runs. UTT is implemented as annual screening of the adult population, and immediate ART for all HIV-infected patients. The intervention is scaled up linearly, starting in 2012 and reaching 90% coverage in 2019 (similar to Granich et al. [9]). The vertical dotted lines give the timing of the start of the intervention. The horizontal dotted lines in the right panels indicate the elimination phase, defined as incidence below 1/1,000 person-years. The same figures for the four main models are given in Figure 2.
(TIF)

Figure S7 **Model fit compared to data (model D).** (A) Projected demographic structure of South Africa in 2011: model compared to UN data [77]. (B) Age-specific HIV prevalence (model compared to data [78]) in 2008. (C) Age-specific distribution in number of partners in the last 12 mo for men. (D) Age-specific distribution in number of partners in the last 12 mo for women. (E) Trend in prevalence of STIs in men: model compared to estimates from Johnson et al. [79]. (F) Trend in prevalence of STIs in women: model compared to estimates from Johnson et al. [79]. (G) Projected ART coverage in South Africa: model versus WHO data [53]. (H) Projected total number of people on ART in South Africa: model versus WHO data [53]. (I) Cumulative distribution of CD4 cell counts at first HIV test: model compared to data from KwaZulu-Natal, South Africa [25],[80]. (J) Average remaining life expectancy at treatment initiation by CD4 cell count at treatment initiation: model compared to data [51].
(TIF)

Figure S8 **Predicted HIV prevalence for all sensitivity analyses (except for alternative economic and UTT performance assumptions) compared to UNAIDS-reported HIV prevalence in South Africa** [**1**] **.** Parameter assumptions are given in Tables S6 and S7.
(TIF)

Table S1 **Annual probability of a woman having a child, by age group.** Distribution over age groups according to UN data [39].
(DOCX)

Table S2 **Sexual behavior parameters.** Justification for the age distribution in promiscuity, frequency of contact, and duration of partnerships can be found in Orroth et al. [35] and Korenromp et al. [30]. All age-specific promiscuity values (i.e., overall partner change rates) were adjusted with the same factor in order to represent the HIV epidemic observed in South Africa.
(DOCX)

Table S3 **Age preference matrix for men and women.** Same as in previous STDSIM studies [25],[30],[35]. Justification for the values can be found in Korenromp et al. [30].
(DOCX)

Table S4 **Parameter settings for commercial sex.** Same as in previous STDSIM studies [25],[35]. Justification can be found in Orroth et al. [35].
(DOCX)

Table S5 **Parameter settings for natural history of simulated STIs.** Same as previously used in other studies [25],[35]. “Lack of circumcision” effect represents factor increase in susceptibility to the STI for men who are not circumcised, i.e., uncircumcised men are twice as likely to get infected during an unprotected sex act with an infected partner. Justification can be found in Orroth et al. [35].
(DOCX)

Table S6 **Alternative assumptions on HIV natural history and transmission probabilities for sensitivity analyses.** Infectiousness in the asymptomatic stage, condom use, and the year of HIV introduction are used to fit predicted HIV prevalence to the UNAIDS data [1].
(DOCX)

Table S7 **Input parameters for sensitivity analysis on the course of the epidemic.**
(DOCX)

Table S8 **Results of the sensitivity analysis.** The year of elimination is defined as the first year HIV incidence drops below 1/1,000 person-years. UTT is universal testing of individuals aged 15+ y and immediate treatment for all HIV-infected patients, starting in 2012 and scaled up to 90% coverage in 2019.
(DOCX)

Text S1 **Sensitivity analysis.**
(DOCX)

Text S2 **Model fit to data.**
(DOCX)

## Abbreviations

ART: antiretroviral therapy
FSW: female sex worker
STI: sexually transmitted infection
UNAIDS: Joint United Nations Programme on HIV/AIDS
UTT: universal test and treat
WHO: World Health Organization
