# Supplementary figures and images for: Elimination of HIV in South Africa through Expanded Access to Antiretroviral Therapy: A Model Comparison Study
Source: PLoS Med. 2013 Oct 22;10(10):e1001534. doi: 10.1371/journal.pmed.1001534 (PMC3805487; doi:10.1371/journal.pmed.1001534)

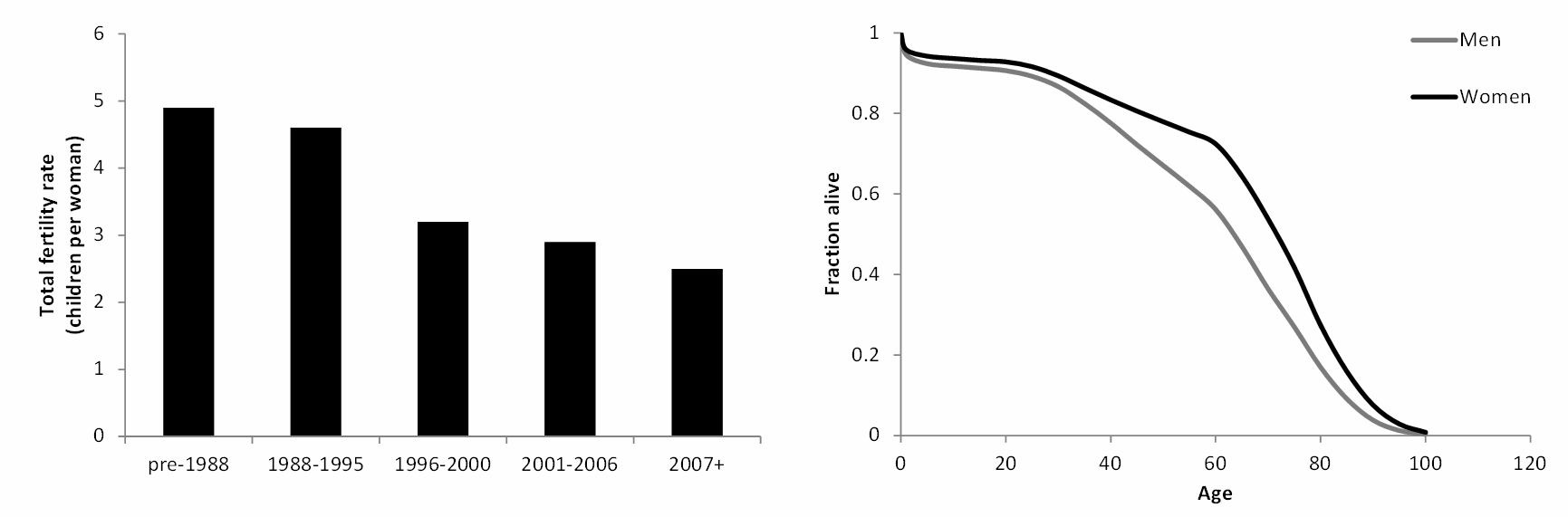

Supplement: Figure S1 — Demographic input parameters. (A) Total fertility rates (lifetime number of births/woman) over time. Data obtained from the UN World Fertility Data 2008 database [39]. (B) Background mortality (mortality in the absence of AIDS) obtained from WHO [40]. All cause mortality rates were corrected using WHO burden of disease estimates of HIV mortality rates in South Africa [41] in order to obtain estimates of non-AIDS-related mortality rates in South Africa. (TIF) [file pmed.1001534.s001.tif]
