# Supplementary material for: Elimination of HIV in South Africa through Expanded Access to Antiretroviral Therapy: A Model Comparison Study
Source: PLoS Med. 2013 Oct 22;10(10):e1001534. doi: 10.1371/journal.pmed.1001534 (PMC3805487; doi:10.1371/journal.pmed.1001534)
Supplement: Table S1 — Annual probability of a woman having a child, by age group. Distribution over age groups according to UN data [39]. (DOCX) [file pmed.1001534.s009.docx]

Table S1. Annual probability for a woman to have a child, by age group**.** Distribution over age groups according to UN data [39].

| **Age group** | **Annual probability of getting a child** |
| --- | --- |
| 15-19 | 0.08 |
| 20-24 | 0.21 |
| 25-29 | 0.23 |
| 30-34 | 0.21 |
| 35-39 | 0.16 |
| 40-44 | 0.06 |
| 45-49 | 0.04 |
