# Supplementary material for: Elimination of HIV in South Africa through Expanded Access to Antiretroviral Therapy: A Model Comparison Study
Source: PLoS Med. 2013 Oct 22;10(10):e1001534. doi: 10.1371/journal.pmed.1001534 (PMC3805487; doi:10.1371/journal.pmed.1001534)
Supplement: Table S2 — Sexual behavior parameters. Justification for the age distribution in promiscuity, frequency of contact, and duration of partnerships can be found in Orroth et al. [35] and Korenromp et al. [30]. All age-specific promiscuity values (i.e., overall partner change rates) were adjusted with the same factor in order to represent the HIV epidemic observed in South Africa. (DOCX) [file pmed.1001534.s010.docx]

Table S2. Sexual behavior parameters. Justification for the age distribution in promiscuity, frequency of contact, and duration of partnerships can be found in Orroth *et al* [35] and Korenromp *et al* [30]. All age specific promiscuity values (i.e. overall partner change rates) were adjusted with the same factor in order to represent the HIV epidemic observed in South Africa.

* determined by the age of the male partner

|  | **Women** | **Men** | **Distribution** |
| --- | --- | --- | --- |
| *Age of sexual debut* | 17  [Range: 15 - 19] | 17  [Range: 15 - 19] | Uniform |
|  |  |  |  |
| *Average time till availability* ($\tau_{s,r}$) |  |  |  |
| Single | 0.5 years | 0.5 years | Exponential |
| Steady relationship | 25 years | 10 years | Exponential |
| Casual relationship | 3.5 years | 2 years | Exponential |
|  |  |  |  |
| *Time to find* (δ) | 0.25 years | 2.25 years | Exponential |
|  |  |  |  |
| *Mean personal promiscuity (p_m_)* | 1.0 | 1.0 | Gamma  (shape = 1.5) |
| *Age specific promiscuity (*$r_{s,a}$*)* |  |  |  |
| 15-19 | 7.8 | 1.9 | Fixed value |
| 20-24 | 7.8 | 3.9 | Fixed value |
| 25-29 | 4.9 | 5.8 | Fixed value |
| 30-34 | 2.9 | 7.8 | Fixed value |
| 35-39 | 1.9 | 4.9 | Fixed value |
| 40-44 | 1.9 | 1.9 | Fixed value |
| 45-49 | 1.9 | 1.9 | Fixed value |
| 50+ | 1.9 | 1.9 | Fixed value |
|  |  |  |  |
| *Probability of steady relationship by age group* |  |  |  |
| 15-19 | N/A* | 0.05 | Fixed value |
| 20-24 | N/A* | 0.1 | Fixed value |
| 25-29 | N/A* | 0.3 | Fixed value |
| 30-34 | N/A* | 0.5 | Fixed value |
| 35-39 | N/A* | 0.7 | Fixed value |
| 40-44 | N/A* | 0.9 | Fixed value |
| 45-49 | N/A* | 0.9 | Fixed value |
| 50+ | N/A* | 0.9 | Fixed value |
|  |  |  |  |
| *Frequency of sexual contact* |  |  |  |
| *Steady relation* |  |  |  |
| 15-34 | N/A* | 6.5 times/month | Exponential |
| 35-49 | N/A* | 4.4 times/month | Exponential |
| 50+ | N/A* | 3.7 times/month | Exponential |
| *Casual relation* |  |  |  |
| 15-19 | N/A* | 3.3 times/month | Exponential |
| 20-24 | N/A* | 2.2 times/month | Exponential |
| 25-29 | N/A* | 1.7 times/month | Exponential |
|  |  |  |  |
| *Average relationship duration* |  |  |  |
| Casual relationship | 0.5 years | 0.5 years | Exponential |
| Steady relationship | 25 years | 25 years | Exponential |
