# Supplementary material for: Elimination of HIV in South Africa through Expanded Access to Antiretroviral Therapy: A Model Comparison Study
Source: PLoS Med. 2013 Oct 22;10(10):e1001534. doi: 10.1371/journal.pmed.1001534 (PMC3805487; doi:10.1371/journal.pmed.1001534)
Supplement: Table S3 — Age preference matrix for men and women. Same as in previous STDSIM studies [25],[30],[35]. Justification for the values can be found in Korenromp et al. [30]. (DOCX) [file pmed.1001534.s011.docx]

Table S3. Age preference matrix for men and women**.** Same as in previous STDSIM studies[25, 35]. Justification for the values can be found in Korenromp *et al* [35]

| Male age (y) |  | Female age (y) | | | | | | | | |  |
| --- | --- | --- | --- | --- | --- | --- | --- | --- | --- | --- | --- |
|  | <15 | | 15-19 | 20-24 | 25-29 | 30-34 | 35-39 | 40-44 | 45-49 | 50+ | |
| <15 | 0.95 | | 0.05 | 0.0 | 0.0 | 0.0 | 0.0 | 0.0 | 0.0 | 0.0 | |
| 15-19 | 0.9 | | 0.1 | 0.0 | 0.0 | 0.0 | 0.0 | 0.0 | 0.0 | 0.0 | |
| 20-24 | 0.7 | | 0.25 | 0.05 | 0.0 | 0.0 | 0.0 | 0.0 | 0.0 | 0.0 | |
| 25-29 | 0.5 | | 0.25 | 0.2 | 0.05 | 0.0 | 0.0 | 0.0 | 0.0 | 0.0 | |
| 30-34 | 0.3 | | 0.25 | 0.2 | 0.2 | 0.05 | 0.0 | 0.0 | 0.0 | 0.0 | |
| 35-39 | 0.1 | | 0.2 | 0.25 | 0.2 | 0.2 | 0.05 | 0.0 | 0.0 | 0.0 | |
| 40-44 | 0.0 | | 0.1 | 0.2 | 0.25 | 0.2 | 0.2 | 0.05 | 0.0 | 0.0 | |
| 45-49 | 0.0 | | 0.0 | 0.1 | 0.2 | 0.25 | 0.2 | 0.2 | 0.05 | 0.0 | |
| 50+ | 0.0 | | 0.0 | 0.0 | 0.1 | 0.2 | 0.25 | 0.2 | 0.2 | 0.05 | |
| Female age (y) |  | Male age (y) | | | | | | | | |  |
|  | <15 | | 15-19 | 20-24 | 25-29 | 30-34 | 35-39 | 40-44 | 45-49 | 50+ | |
| <15 | 0.1 | | 0.3 | 0.3 | 0.25 | 0.05 | 0.0 | 0.0 | 0.0 | 0.0 | |
| 15-19 | 0.0 | | 0.15 | 0.4 | 0.3 | 0.1 | 0.05 | 0.0 | 0.0 | 0.0 | |
| 20-24 | 0.0 | | 0.0 | 0.15 | 0.4 | 0.3 | 0.1 | 0.05 | 0.0 | 0.0 | |
| 25-29 | 0.0 | | 0.0 | 0.0 | 0.15 | 0.4 | 0.3 | 0.1 | 0.05 | 0.0 | |
| 30-34 | 0.0 | | 0.0 | 0.0 | 0.0 | 0.15 | 0.4 | 0.3 | 0.15 | 0.0 | |
| 35-39 | 0.0 | | 0.0 | 0.0 | 0.0 | 0.0 | 0.15 | 0.4 | 0.4 | 0.05 | |
| 40-44 | 0.0 | | 0.0 | 0.0 | 0.0 | 0.0 | 0.0 | 0.15 | 0.7 | 0.15 | |
| 45-49 | 0.0 | | 0.0 | 0.0 | 0.0 | 0.0 | 0.0 | 0.05 | 0.8 | 0.15 | |
| 50+ | 0.0 | | 0.0 | 0.0 | 0.0 | 0.0 | 0.0 | 0.0 | 0.05 | 0.95 | |
