# Supplementary material for: Elimination of HIV in South Africa through Expanded Access to Antiretroviral Therapy: A Model Comparison Study
Source: PLoS Med. 2013 Oct 22;10(10):e1001534. doi: 10.1371/journal.pmed.1001534 (PMC3805487; doi:10.1371/journal.pmed.1001534)
Supplement: Table S4 — Parameter settings for commercial sex. Same as in previous STDSIM studies [25],[35]. Justification can be found in Orroth et al. [35]. (DOCX) [file pmed.1001534.s012.docx]

Table S4. Parameter settings for commercial sex. Same as in previous STDSIM studies [25, 35]. Justification can be found in Orroth *et al* [35].

| **Female sex workers** |  |
| --- | --- |
| Start age | 17-30 years |
| Max. stop age | 35 years |
| Minimum career length | 1 year |
|  |  |
| **Clients** |  |
| Proportion of men by frequency |  |
| Married |  |
| 0 visits/year | 67% |
| 1 visit/year | 28% |
| 12 visits/year | 5% |
| Unmarried |  |
| 0 visits/year | 34% |
| 1 visit/year | 55% |
| 12 visits/year | 11% |
