# Supplementary material for: Elimination of HIV in South Africa through Expanded Access to Antiretroviral Therapy: A Model Comparison Study
Source: PLoS Med. 2013 Oct 22;10(10):e1001534. doi: 10.1371/journal.pmed.1001534 (PMC3805487; doi:10.1371/journal.pmed.1001534)
Supplement: Table S5 — Parameter settings for natural history of simulated STIs. Same as previously used in other studies [25],[35]. “Lack of circumcision” effect represents factor increase in susceptibility to the STI for men who are not circumcised, i.e., uncircumcised men are twice as likely to get infected during an unprotected sex act with an infected partner. Justification can be found in Orroth et al. [35]. (DOCX) [file pmed.1001534.s013.docx]

Table S5. Parameter settings for natural history of simulated STDs. Same as previously used in other studies [25,35]. 'Lack of circumcision' effect represents factor increase in susceptibility to the STD for men who are not circumcised, i.e. uncircumcised men are twice as likely to get infected during an unprotected sex-act with an infected partner. Justification can be found in Orroth *et al* [35].

^1^Recurrent ulcers occur during the late latent stage of HSV-2. Average duration between recurrent ulcers is 3 months in early-latent, and 8 months in late-latent.

|  | **Duration** |  | **Transmission probabilities** | |  | **Cofactor effect on HIV transmission** | **'Lack of circumcision' effect** |
| --- | --- | --- | --- | --- | --- | --- | --- |
|  |  | **M-->F** | | **F-->M** | |  |  |
| Chancroid | 11 weeks | 0.23 | | 0.115 | | 25 | 2 |
|  |  |  | |  | |  |  |
| Gonorrhea | 14 weeks | 0.26 | | 0.13 | | 3 | 1 |
|  |  |  | |  | |  |  |
| Chlamydia | 14 weeks(M); 52 weeks (F) | 0.252 | | 0.126 | | 3 | 1 |
|  |  |  | |  | |  |  |
| Syphilis |  |  | |  | |  | 2 |
| Primary | 6 months | 0.175 | | 0.088 | | 7.5 |  |
| Early latent | 1 year | 0.018 | | 0.009 | | 1 |  |
| Latent | 2.5 years | 0 | | 0 | | 1 |  |
| Late latent | 12.5 years | 0 | | 0 | | 1 |  |
| HSV-2 |  |  | |  | |  | 2 |
| Primary | 3 weeks | 0.3 | | 0.15 | | 25 |  |
| Early latent  (with recurrent ulcer) | 2 years | 0.01 | | 0.005 | | 1 |  |
| Late latent  (with recurrent ulcer) | 10 years | 0.005 | | 0.003 | | 1 |  |
| Late latent | Lifelong | 0 | | 0 | | 1 |  |
| Recurrent ulcer^1^ | 1 week | 0.2 | | 0.1 | | 10 |  |
