# Supplementary material for: Elimination of HIV in South Africa through Expanded Access to Antiretroviral Therapy: A Model Comparison Study
Source: PLoS Med. 2013 Oct 22;10(10):e1001534. doi: 10.1371/journal.pmed.1001534 (PMC3805487; doi:10.1371/journal.pmed.1001534)
Supplement: Table S6 — Alternative assumptions on HIV natural history and transmission probabilities for sensitivity analyses. Infectiousness in the asymptomatic stage, condom use, and the year of HIV introduction are used to fit predicted HIV prevalence to the UNAIDS data [1]. (DOCX) [file pmed.1001534.s014.docx]

|  | **Model D**  **(STDSIM)** | **'Powers' parameterization** | **'Williams' parameterization** | **Model D (STDSIM) - No transmission increase during symptomatic infection** | **Model D (STDSIM) -**  **Reduce co-factor effects of other STIs by 2/3** |
| --- | --- | --- | --- | --- | --- |
| **Infectiousness relative to asymptomatic stage** |  |  |  |  |  |
| Acute | 15 | 30.3 | 3.2 | 15 | 15 |
| Asymptomatic | 1 | 1 | 1 | 1 | 1 |
| Symptomatic | 3 | 3 | 3 | 1 | 3 |
| AIDS | 7.5* | 7.5** | 7.5* | 7.5* | 7.5* |
| **Average duration** |  |  |  |  |  |
| Acute | 3 months | 4.8 months | 2 weeks | 3 months | 3 months |
| Asymptomatic | 5 years | 5.7 years | 5 years | 5 years | 5 years |
| Symptomatic | 4 years | 1.1 year | 4 years | 4 years | 4 years |
| AIDS | 8 months | 1.1 year | 8 months | 8 months | 8 months |
|  |  |  |  |  |  |
| **Infectiousness asymptomatic stage** | 0.00095 | 0.00065 | 0.0013 | 0.0011 | 0.0014 |
|  |  |  |  |  |  |
| **Condom use** |  |  |  |  |  |
| 1999 | 10% | 5% | 10% | 10% | 10% |
| 2000 | 20% | 10% | 20% | 20% | 20% |
| 2002 onward | 30% | 15% | 30% | 30% | 30% |
|  |  |  |  |  |  |
| **Year of HIV introduction** | 1988 | 1992 | 1979 | 1988 | 1988 |
|  |  |  |  |  |  |

Table S6. Alternative assumptions on HIV natural history and transmission probabilities for sensitivity analyses. Infectiousness in the asymptomatic stage, condom use, and the year of HIV introduction are used to fit predicted HIV prevalence to the UNAIDS data [1].

* Frequency of sexual contact within a relationship is reduced by 50% due to ill health

** Frequency of sexual contact within a relationship is reduced by 100% due to ill health
