# Supplementary material for: Elimination of HIV in South Africa through Expanded Access to Antiretroviral Therapy: A Model Comparison Study
Source: PLoS Med. 2013 Oct 22;10(10):e1001534. doi: 10.1371/journal.pmed.1001534 (PMC3805487; doi:10.1371/journal.pmed.1001534)
Supplement: Table S7 — Input parameters for sensitivity analysis on the course of the epidemic. (DOCX) [file pmed.1001534.s015.docx]

|  | **Model D (STDSIM)** | **Increasing prevalence in 2000s** | **Alternative behavior change assumptions** |
| --- | --- | --- | --- |
| **Condom use** |  |  |  |
| 1999 | 10% | 5% | 5% |
| 2000 | 20% | 10% | 10% |
| 2002 onward | 30% | 15% | 15% |
| **Reduction in promiscuity^1^** |  |  |  |
| 1999 | N/A | N/A | 90% |
| 2000 | N/A | N/A | 75% |
| **Proportion of men (aged 15-49) with 2+ partners ^2^** |  |  |  |
| 1998 | 41% | 41% | 41% |
| 2012 | 41% | 41% | 35% |

#### **Table S7**. Input parameters for sensitivity analysis on the course of the epidemic

^1^ Level of *age- and sex-specific promiscuity* relative to the baseline. Baseline values are given in table S1 and explained in section 2.3.1

^2^ Over the last 12 months. To illustrate the effect of the adjustment in the *age- and sex-specific promiscuity*.
