# Supplementary material for: Elimination of HIV in South Africa through Expanded Access to Antiretroviral Therapy: A Model Comparison Study
Source: PLoS Med. 2013 Oct 22;10(10):e1001534. doi: 10.1371/journal.pmed.1001534 (PMC3805487; doi:10.1371/journal.pmed.1001534)
Supplement: Table S8 — Results of the sensitivity analysis. The year of elimination is defined as the first year HIV incidence drops below 1/1,000 person-years. UTT is universal testing of individuals aged 15+ y and immediate treatment for all HIV-infected patients, starting in 2012 and scaled up to 90% coverage in 2019. (DOCX) [file pmed.1001534.s016.docx]

Table S8. Results of the sensitivity analysis. The year of elimination is defined as the first year HIV incidence drops below 1/1,000 person-years. UTT = universal testing and immediate treatment for all HIV infected patients, starting in 2012 and scaled up to 90% coverage in 2019.

^1^ Stage of the HIV infection in the index case of an HIV transmission event in the year 2003 (year prior to introduction of ART in the model)

|  |  | **Stage of infection of the index case^1^** | |  |  | **Year of HIV 'elimination'** | |  | **ICER for UTT** |
| --- | --- | --- | --- | --- | --- | --- | --- | --- | --- |
|  | *Early infection* | | ≤350 cells/µL | | ≤350 cells/µL | | *UTT* | |  |
| **Model D (STDSIM)** | 23% | | 52% | | 2041 | | 2029 | | 170 US$/LYS |
|  |  | |  | |  | |  | |  |
| **Average HIV-negative CD4 cell count**  (baseline = 1116 cells/µL) |  | |  | |  | |  | |  |
| 1674 cells/µL | 23% | | 40% | | 2050 | | 2030 | | 350 US$/LYS |
| 744 cells/µL | 23% | | 65% | | 2035 | | 2028 | | Dominant |
|  |  | |  | |  | |  | |  |
| **Course of the epidemic in South Africa** |  | |  | |  | |  | |  |
| Increasing prevalence in 2000s | 23% | | 51% | | >2075 | | 2062 | | Dominant |
| Alternative behavior change assumptions | 23% | | 51% | | 2039 | | 2027 | | 220 US$/LYS |
|  |  | |  | |  | |  | |  |
| **Alternative HIV transmission parameterization** |  | |  | |  | |  | |  |
| 'Powers' parameterization | 47% | | 38% | | No elimination | | No elimination | | Dominant |
| 'Williams' parameterization | 2% | | 62% | | 2047 | | 2032 | | Dominant |
| No transmission increase during symptomatic infection | 31% | | 39% | | 2050 | | 2031 | | 20 US$/LYS |
| Reduce co-factor effects of other STIs by 2/3 | 22% | | 55% | | 2043 | | 2032 | | 200 US$/LYS |
|  |  | |  | |  | |  | |  |
| **Alternative economic assumptions** |  | |  | |  | |  | |  |
| Discount rate (baseline = 3%) |  | |  | |  | |  | |  |
| *10%* | 23% | | 52% | | 2041 | | 2029 | | 1 200 US$/LYS |
| *1%* | 23% | | 52% | | 2041 | | 2029 | | 53 US$/LYS |
| Scale effects |  | |  | |  | |  | |  |
| *Economies of scale* | 23% | | 52% | | 2041 | | 2029 | | 850 US$/LYS |
| *Diseconomies of scale* | 23% | | 52% | | 2041 | | 2029 | | 1 100 US$/LYS |
|  |  | |  | |  | |  | |  |
| Costs for Infrastructural expansion | 23% | | 52% | | 2041 | | 2029 | | 624 US$/LYS |
|  |  | |  | |  | |  | |  |
| No differentiation by CD4 at initiation and years on treatment | 23% | | 52% | | 2041 | | 2029 | | 960 US$/LYS |
|  |  | |  | |  | |  | |  |
| **Less optimistic programmatic assumptions** |  | |  | |  | |  | |  |
| 60% annual screening coverage | 23% | | 52% | | 2041 | | 2031 | | 132 US$/LYS |
| 5% annual rate of stopping treatment, 8.5% in first year | 23% | | 52% | | 2041 | | 2048 | | Dominated |
