# Supplementary material for: Elimination of HIV in South Africa through Expanded Access to Antiretroviral Therapy: A Model Comparison Study
Source: PLoS Med. 2013 Oct 22;10(10):e1001534. doi: 10.1371/journal.pmed.1001534 (PMC3805487; doi:10.1371/journal.pmed.1001534)
Supplement: Text S1 — Sensitivity analysis. (DOCX) [file pmed.1001534.s017.docx]

**Text S1: Sensitivity analysis**

We performed a sensitivity analysis on the predicted year of HIV elimination under UTT and continued scale-up of ART at ≤350 cells/µL (figure 2 in the main text), and the cost-effectiveness of UTT compared to continued scale-up of ART at ≤350 cells/µL (table 2 in the main text) in *model D*. We varied parameters on HIV natural history and transmission dynamics, the course of the HIV epidemic in South Africa, and economics of ART. For each alternative setting (except when assuming a different course of the HIV epidemic), we re-fitted the model predictions in order to make the predicted HIV prevalence comparable to the baseline (*model D* - see figure 2 in the main text).

## 1.1 Parameter settings

### 1.1.1 HIV natural history and transmission dynamics

First, we increase and decrease the HIV negative CD4 cell count distribution through multiplication by a factor 3/2nd (median HIV negative CD4 cell count of 1674) and 2/3rd (median CD4 cell count of 744) respectively. Second, the contribution of different stages of the HIV infection (e.g. early infection versus late infection) to the overall HIV transmission is uncertain and under intense debate [17, 58, 81]. Therefore, we assume the following alternative parameterizations of HIV disease progression and transmission: (i) 'Powers' parameterization (assumptions based on Powers *et al* [17]); (ii) 'Williams' parameterization (assumptions based on Williams *et al* [81]); (iii) no increase in transmission probabilities in the *symptomatic infection* stage; and (iv) co-cofactor effects of the STDs decrease by a factor 2/3. The predicted HIV prevalence will change due to these alternative assumptions. Therefore, we used the infectiousness in the asymptomatic stage, year of HIV introduction, and condom use rates to fit the predicted HIV prevalence to again represent UNAIDS reported data (figure S8). Table S6 gives all parameter values for each of the 4 scenarios and the baseline.

### 1.1.2 Course of the epidemic

UNAIDS reported HIV prevalence and incidence data depend on model based extrapolations from cross-sectional surveys, and some argue that their reported decline in incidence and thus reported steady state in HIV prevalence might not be true given observations in population based cohorts [82], while others say that the decline in incidence is the result of a combination of an increase in condom use together with a reduction in number of partners. Therefore, we have also performed a sensitivity analysis on the predicted trend in the HIV epidemic by: (i) reducing condom use uptake by half, thus resulting in an increasing rather than a stabilizing HIV epidemic in 2000-2010; and (ii) the reduction in incidence is achieved through a combination of increased condom use and decreased partner change rates. We decrease partner change rates by proportionally reducing all age- and sex-specific promiscuity levels (*see 2.3.1 heterogeneity in sexual behavior*). Parameter assumptions and the resulting change in sexual behavior are given in table S7. Figure S8 gives the fit compared to the UNAIDS reported data [1].

### 1.1.3 Alternative economic assumptions

Finally, we addressed alternative economic assumptions: (i) higher and lower discount rates (10% and 1% respectively); (ii) economies and diseconomies of scale (exponentially increase or decrease in per-patient costs respectively when patient loads increase [83]); (iii) a 20% increase in total costs reflecting infrastructural expansion in the 'test-and-treat' scenario; and (iv) no differentiation of annual ART costs by CD4 cell count at initiation and number of years on treatment.

### 1.1.4 Less optimistic programmatic assumptions

The assumed UTT intervention of 90% coverage and 1.5% dropout is very optimistic. It is likely that screening coverage will be lower and dropout rates will be higher. Therefore, we add two additional UTT scenarios to our sensitivity analysis: (i) annual screening coverage of 60% instead of 90%; and (ii) dropout rates of 5% annually instead of 1.5% (and still an initial dropout rate of 8.5% in the first year of treatment).

## 1.2 Results sensitivity analysis

Table S8 shows the results of the sensitivity analysis. Elimination of HIV (incidence <1/1,000 person-years) is achieved in all of the scenarios, except when assuming high proportions of transmissions due to acute infection. The time until elimination is especially affected by the course of the epidemic. If HIV prevalence in South Africa in the 2000s would continue to rise rather than stabilize, elimination will only be achieved in 2062 (for UTT) and at around 2100 (for ART at ≤350 cells/µL). For all other scenarios, timing of elimination remains relatively unchanged. UTT is highly cost-effective in all of the scenarios in our sensitivity analysis.
