# Supplementary material for: Elimination of HIV in South Africa through Expanded Access to Antiretroviral Therapy: A Model Comparison Study
Source: PLoS Med. 2013 Oct 22;10(10):e1001534. doi: 10.1371/journal.pmed.1001534 (PMC3805487; doi:10.1371/journal.pmed.1001534)
Supplement: Text S2 — Model fit to data. (DOCX) [file pmed.1001534.s018.docx]

# Text S2: Model fit to data

## 2.1 HIV prevalence fit (all models)

For all main models (*A* to *D*) the fit to UNAIDS reported HIV prevalence in South Africa is given in figure 2 of the main text [1]. In addition, figure S6 gives the fit of all sub-models compared to data, and the predicted impact of UTT on HIV incidence and prevalence (see also table 1 in the main text).

## 2.2 Model fit to other data (*model D*)

Figure S7 shows the predicted demographic structure, sexual behavior, age-specific HIV prevalence, STD prevalence, ART roll-out, and CD4 cell count distributions, and survival of those on treatment by CD4 cell count at initiation in *model D* compared to data. Panel A in figure S7 shows that the projected demographic structure of the population matches closely to the data reported by the United Nations Population Division [77]. Panel B in figure S7 shows that the age-specific HIV prevalence in women matches closely to data from the national HIV surveillance [78]. Age specific distribution in numbers of partners for men and women is given in panels C and D in figure S7 respectively. Although it is hard to compare these trends to data as reliable estimates are scarce, predicted distributions are close to those observed by Johnson *et al* [84]. Their estimates show that 41% of all men aged 15-49 report more than 1 partner in the last twelve months, and 25% of all women aged 15-49 report more than 1 recent partner. In our model, these figures are 42% and 34%. It is not unlikely that women underreport their sexual behavior [85-87], explaining the slightly higher proportion of women with multiple partners in our model. In addition, our projected trends in STDs (panels E and F in figure S7) are comparable to those reported by Johnson *et al* [79], who performed a Bayesian analysis of all sentinel surveillance data to estimate the national South African trends in STD prevalence. Prevalence levels for gonorrhea and chlamydia are higher and lower respectively compared to the estimates form Johnson *et al* [79], yet these data are broad estimates with considerable uncertainty and our estimates still fall within their 95% confidence interval [79]. In addition, our model was able to accurately replicate STD prevalence levels of a rural South African area in KwaZulu-Natal [25]. Both in terms of coverage (panel G in figure S7) and total numbers of people on treatment (panel H in figure S7) over the period 2006-2011, our model predictions match closely to data from WHO [53]. The CD4 cell count distributions in those coming to a clinic for the first time matches closely to observed data (panel I in figure S7) from a large treatment cohort in rural Africa [25,80], indicating a nearly perfect fit for health seeking behavior and ART roll-out in South Africa. Finally, average life-expectancy of those on treatment by CD4 cell count at initiation matches closely to data from a large treatment cohort in KwaZulu-Natal (panel J in figure S7) [51].
